# Unilateral Orbital Metastasis as the Unique Symptom in the Onset of Breast Cancer in a Postmenopausal Woman: Case Report and Review of the Literature

**DOI:** 10.3390/diagnostics11040725

**Published:** 2021-04-19

**Authors:** Cristina Marinela Oprean, Larisa Maria Badau, Nusa Alina Segarceanu, Andrei Dorin Ciocoiu, Ioana Alexandra Rivis, Vlad Norin Vornicu, Teodora Hoinoiu, Daciana Grujic, Cristina Bredicean, Alis Dema

**Affiliations:** 1 Morphopathology Department, “Victor Babeş” University of Medicine and Pharmacy, Eftimie Murgu Sq. Nr.2, 300041 Timişoara, Romania; coprean@yahoo.com (C.M.O.); dema.alis@umft.ro (A.D.); 2 Department of Oncology—ONCOHELP Hospital Timisoara, Ciprian Porumbescu Street, No. 59, 300239 Timisoara, Romania; larisa_badau@yahoo.com (L.M.B.); nusaseg@yahoo.com (N.A.S.); ciocoiuandrei1993@gmail.com (A.D.C.); vornicuvlad91@gmail.com (V.N.V.); 3 Department of Oncology—ONCOMED Outpatient Unit Timisoara, Ciprian Porumbescu Street, No. 59, 300239 Timisoara, Romania; 4 Hygiene Department, “Victor Babeş” University of Medicine and Pharmacy, Eftimie Murgu Sq. No.2, 300041 Timişoara, Romania; 5 Neurosciences Department, “Carol Davila” University of Medicine and Pharmacy of Bucharest, 020021 Bucharest, Romania; ioana.rivis@gmail.com; 6 Neurosurgery Department, “Victor Babeş” University of Medicine and Pharmacy, Eftimie Murgu Sq. Nr.2, 300041 Timişoara, Romania; 7 Department of Clinical Practical Skills, “Victor Babeş” University of Medicine and Pharmacy, Eftimie Murgu Sq. Nr.2, 300041 Timişoara, Romania; 8 Clinic of Burns, Plastic and Reconstructive Surgery, “Pius Branzeu” Emergency County Hospital, 300041 Timisoara, Romania; dcalistru@yahoo.com; 9 Department of Plastic and Reconstructive Surgery, “Victor Babeş” University of Medicine and Pharmacy, Eftimie Murgu Sq. Nr.2, 300041 Timişoara, Romania; 10 Department of Psychiatry, “Victor Babeş” University of Medicine and Pharmacy, Eftimie Murgu Sq. Nr.2, 300041 Timişoara, Romania; bredicean.ana@umft.ro

**Keywords:** breast cancer, postmenopausal, tubulo-lobular carcinoma, orbital metastasis

## Abstract

The orbit represents an unusual metastases site for patients diagnosed with cancer, however, breast cancer is the main cause of metastases at this level. These orbital metastases were discovered in patients with a history of breast cancer as unique or synchronous lesions. We present a rare case of a unique retroocular metastasis as the first initial symptom of a tubulo-lobular mammary carcinoma in a postmenopausal woman. A 57-year-old patient complains of diplopia, diminishing visual acuity, orbital tenderness, slight exophthalmia and ptosis of the left eyelid, with insidious onset. Clinical examination and subsequent investigations revealed a left breast cancer cT2 cN1 pM1 stage IV. Breast conserving surgery was performed on the left breast. Pathological examination with immunohistochemistry staining established the complete diagnostic: pT2pN3aM1 Stage IV breast cancer, luminal B subtype. After two years from the initial breast cancer diagnosis, the patient was diagnosed by the psychiatrist with a depressive disorder and was treated accordingly. Orbital metastases are usually discovered in known breast cancer patients and they are found in the context of a multi-system end-stage disease. Most reports cite that up to 25% of the total orbital metastases cases are discovered before the diagnosis of the primary tumor, as our case did. MRI is the gold standard for evaluating orbital tumors. The ILC histological subtype metastasizes in the orbitals more frequently than invasive ductal carcinoma. The prognosis of patients with orbital metastases is poor. The median survival after diagnosis of orbital metastases from a breast cancer primary is ranging from 22 to 31 months. Overall survival of our patient was 56 months, longer than the median survival reported in literature. Orbital metastases must be taken into account when patients accuse ophthalmologic symptoms even in the absence of a personal history of cancer. Objective examination of every patient that incriminates these types of symptoms is essential, and breast palpation must be made in every clinical setting. Orbital biopsy is necessary for the confirmation of the diagnosis and for an adequate treatment. Although recommendations for management of orbital metastases are controversial, it appears that multidisciplinary treatment of both metastases and primary cancer improves overall survival.

## 1. Introduction

Breast cancer is the most frequent form of cancer amongst women, and, in the most part, the presence of metastases dictates the survival rate. Approximately 6% of women diagnosed with breast cancer have metastases present at the moment of presentation [1,2]. Between 2 and 5% of total diagnosed cancer patients will develop orbital metastases [3]. Breast cancer can metastasize in many organs, but it is responsible for the most frequent orbital metastases (28.5–58.8% of all cases of orbital metastasis) followed by prostate and lung cancer [4]. Invasive lobular carcinoma (ILC) has a higher likelihood to metastasize in unusual sites such as the gastrointestinal tract, genitourinary system, peritoneum, retroperitoneal, bone, bone marrow, central nervous system, leptomeninges, and orbit, whereas invasive ductal carcinoma (IDC) often metastasizes to the lung, liver, bone, and brain [5].

Although the majority of the patients with orbital metastases, male or female, have a history of breast cancer [6], in 25% of the cases, orbital metastases are the first manifestation of this disease [7]. Differentiation between an orbital metastasis and a primary orbital tumor may be complicated by the diverse symptomatology or by the absence of a known breast cancer diagnosis. Establishing the diagnosis of orbital metastases requires the implicit tissue biopsy or, when it is possible, surgical excision of the tumor. 

We present the case of a 57-year-old postmenopausal woman diagnosed with unilateral retroocular metastasis as the first and only symptom of a mammary tubulo-lobular carcinoma. This fact leads us to evaluate the current specialty literature regarding the incidence, clinical presentation, diagnostic modalities, therapeutic management, treatment response, and disease-progression free survival of invasive breast cancer with orbital metastasis. 

## 2. Case Report

A 57-year-old postmenopausal Caucasian country side woman, with no family history of breast or gynecological cancer. She had her menarche at the age of 15, gave birth at the age of 23, breast fed for two years, never used hormonal contraception accused the following since March 2015: diplopia, diminished visual acuity, orbital tenderness, mild exophthalmia of her left eye and ptosis of the left eyelid, all debuting insidiously. Furthermore, the patient has several comorbidities such as diabetes mellitus type 2, essential arterial hypertension, and mixed cardiomyopathy.

**Figure 1:**
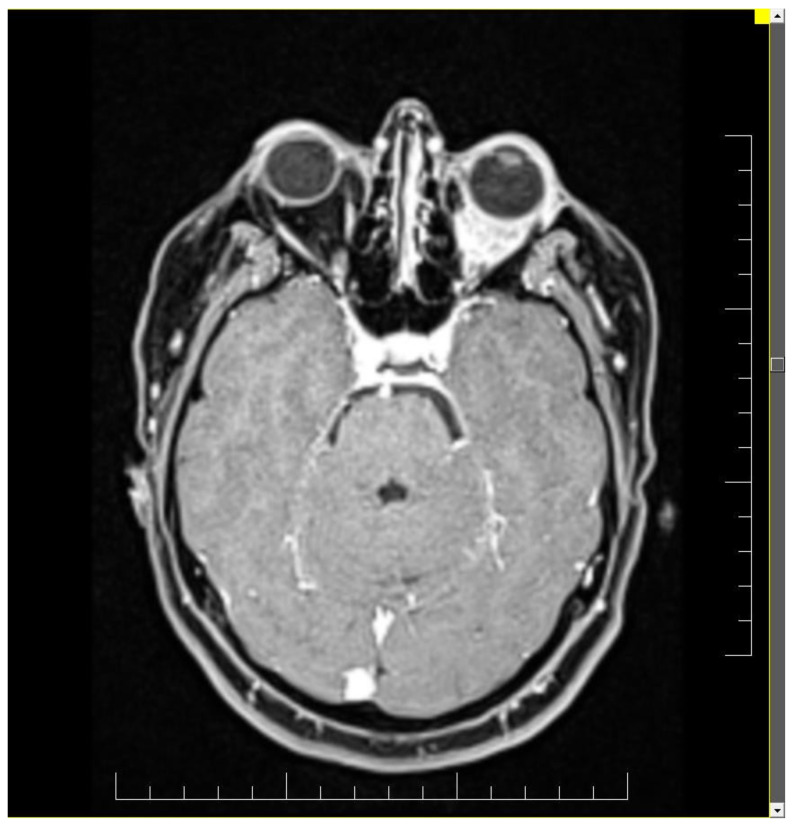
Brain CT scan showing an inhomogeneous mass with contrast agent capture in the retroocular space of the left orbit, cuffing the left optic nerve.

**Figure 2:**
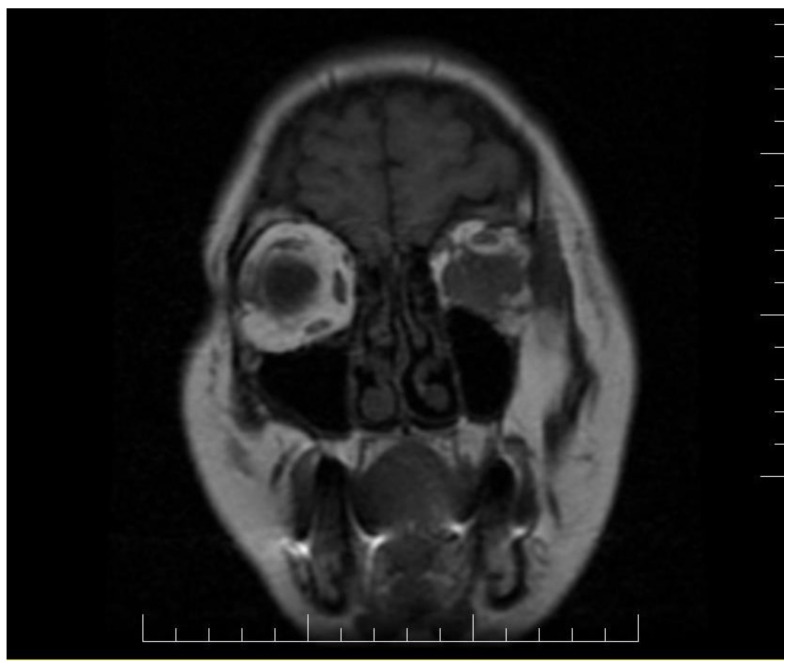
Brain MRI exhibiting a left retroocular intraorbital tumor, which infiltrated the ocular muscles in three out of quarter quadrants.

Ophthalmologic and neurologic examinations revealed reduced visual acuity, reduced mobility in all directions of the eye, normal intraocular pressure, hypoesthesia of the left hemi-face, paresis of the 3rd and 4th nerve of the left eye and paresis of the 5th nerve. Magnetic resonance imaging (MRI) and computed tomography (CT) scan detect a tumor situated in the left retroocular space in the intraconal area, which cuffs the left optic nerve and invades the extra-ocular muscles (Figure 1 and Figure 2). Differential diagnosis included diabetic retinopathy, thyroid ophthalmopathy, lymphoma, primary or metastatic neoplasia. The thyroid function tests were normal and the ophthalmologic exam excluded diabetic retinopathy.

In June 2015 an excisional biopsy of the tumor was made and the pathological report confirmed the metastasis of an invasive ductal breast cancer. Immunohistochemistry (IHC) analysis revealed carcinoembryonic antigen, synaptophysin, cytokeratin 20 negativity and cytokeratin (CK) 7, estrogen receptor (ER) positivity.

**Figure 3:**
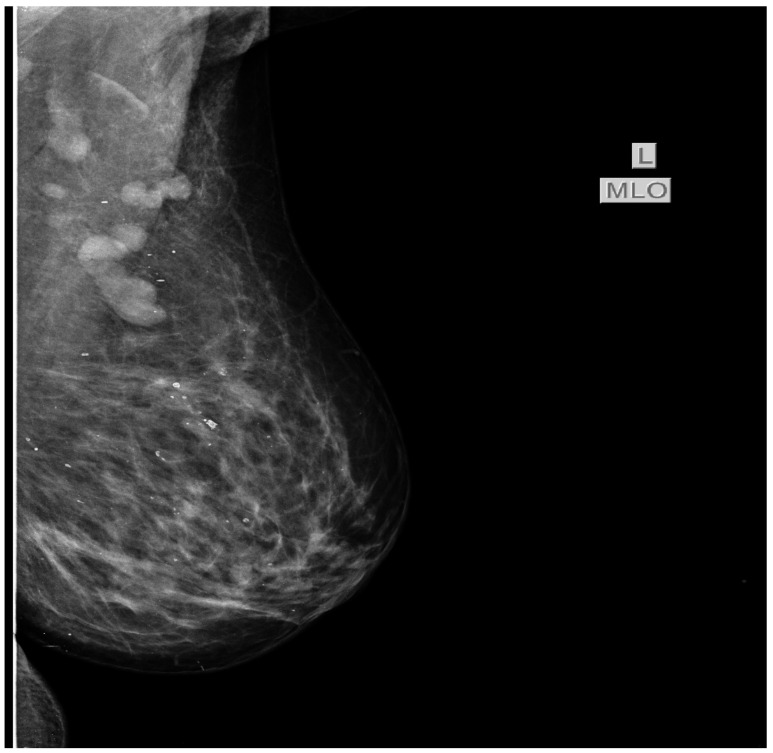
Bilateral mammography shows in the upper external quadrant of the left breast a density asymmetry, with multiple microcalcifications and associate multiple axillary adenopathies without the visualization of the fatty hilum.

**Figure 4:**
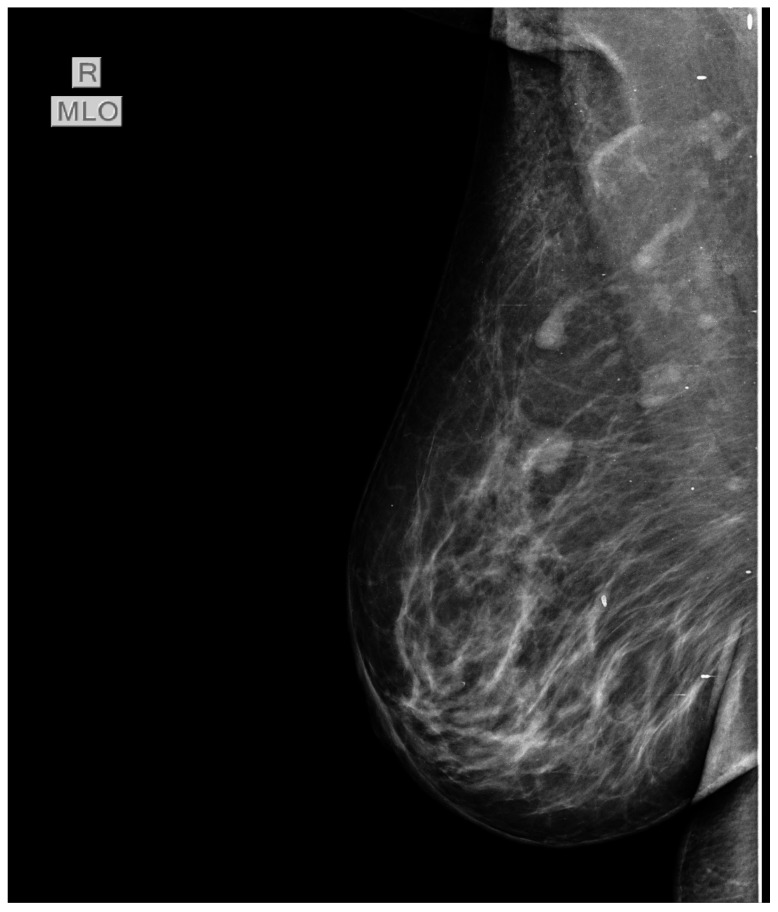
Mammography also revealed in the upper internal quadrant of the right breast, an oval shaped image, well circumscribed, with benign characteristics suggestive for a fibroadenoma.

In July 2015 the patient was referred to our oncology clinic for management of an orbital metastasis with primary breast cancer. She was in a good general health status, had normal weight and Eastern Cooperative Oncology Group (ECOG) 0. Clinical examination of the breasts revealed a palpable mass of 2.5/3 cm in size in the left upper outer quadrant and the enlargement of ipsilateral axillary lymph nodes (cN1). In the upper internal quadrant of the right breast a tumor of 1.5/1 cm with a firm consistency and a smooth surface is palpated. The value of carbohydrate antigen 15-3 (CA 15-3) was 53 U/mL (normal values < 28 U/mL). Subsequently, standard mammography and bilateral breast echography were performed. Mammography showed in the upper external quadrant of the left breast a density asymmetry, with multiple micro-calcifications and associates multiple axillary adenopathies without the visualization of the fatty hilum (Figure 3). On the upper internal quadrant of the right breast, mammography also revealed an oval shaped image with benign characteristics suggestive for a fibroadenoma (Figure 4). Breast ultrasound revealed the left breast discerned an inhomogeneous hypoechogenic area of 23/22 mm, imprecisely delimited in the upper external quadrant and ipsilateral adenopathies. Breast imaging reporting and data system score (BIRADS) was 5 for the left breast, suggestive of malignancy. For the right breast, the BIRADS score was 2 with a suggestive aspect of mammary fibroadenoma. The established clinical diagnosis for this stage was: left superior external quadrant breast cancer cT2 cN1 pM1 stage IV.

**Figure 5:**
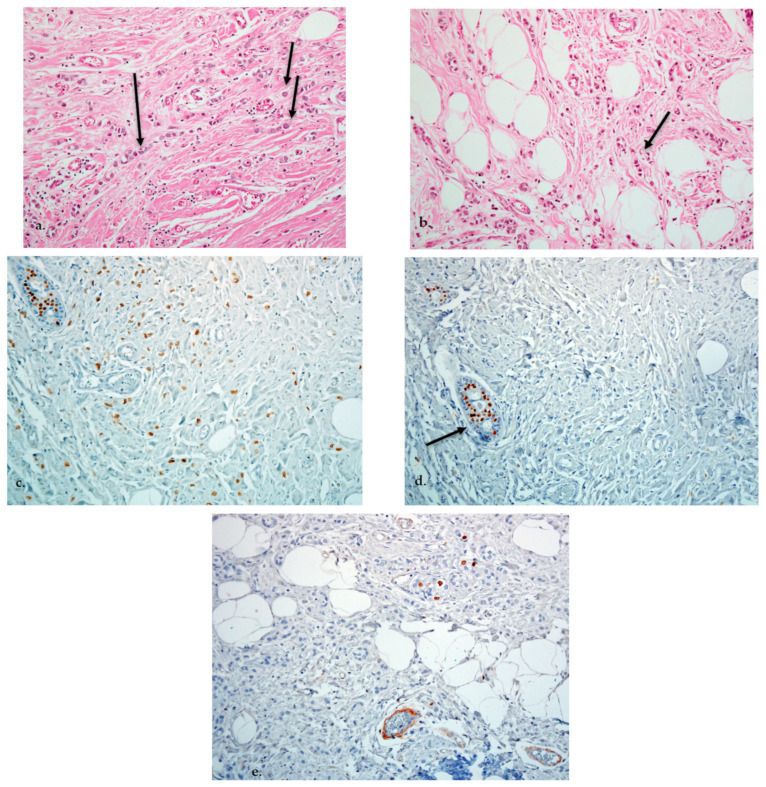
(**a**) Invasive lobular carcinoma: isolated tumor cells and “indian file” arrangement (arrows) (H and E staining 20×)**.** (**b**) Some invasive tumor cells have signet-ring cell morphology (arrow) (H and E staining 20×)**.** (**c**) High expression of estrogen receptor (ER) in the nuclei of tumor cells. 20×. (**d**) Absence of progesterone receptor (PR) in the tumor cells (internal positive control−benign glands−arrow) × 20. Anti-PR 20×. (**e**) Low proliferative activity. Anti-Ki-67 20×.

In July 2015 surgical treatment is performed on the left breast tumor: sectorial mastectomy with ipsilateral lymphadenectomy. Pathological examination revealed a grade 2 invasive carcinoma, tubulo-lobular subtype with lympho-vascular invasion, perineural invasion and positive margins, 10 positive axillary lymph nodes out of 10 examined, pT2pN3a—according to the 7th edition of the Union of International Cancer Control (UICC) Tumor, Nodes, Metastasis (TNM) classification. Furthermore, IHC staining showed ER—positive (67%), PR—negative, human epidermal growth factor receptor 2 (HER2/neu) negative and Ki-67 index of 9% (luminal B subtype). Post-surgery total body CT scan detects no other distant metastases. (Figure 5).

In September 2015, the patient was referred to the radiotherapy department for external beam radiation therapy. The residual left orbital mass was irradiated with 50 Grays (Gy) in 25 fractions on the left eye. Concomitantly, hormonal therapy with aromatase inhibitors was initiated: Anastrozole 1 mg o.d. (once a day), daily. Additionally, she underwent further radiation therapy on the chest wall, breast and internal mammary lymph nodes (total dose 50 Gy/25 fractions) and left axillary and left supraclavicular nodes (total dose 50 Gy/20 fractions).

After radiation therapy was finalized, the patient was followed up according to the 2015 European Society of Medical Oncology (ESMO) recommendations.

After two years from the initial breast cancer diagnosis, the patient developed a depression, anhedonia, hypochondriac preoccupations, memory and concentration disorders, anxiety and mixt insomnia reason why she was referred to a psychiatrist. She was diagnosed with a depressive disorder for which she was prescribed citalopramum 20 mg o.d. and alprazolamum 0.5 mg t.i.d (three times a day).

At a follow-up control from February 2019 the patient is asymptomatic with the exception of unilateral blindness secondary to irradiation. ECOG performance status was 0. Biologically, CA15-3 tumor marker value was increased at 4339 U/mL, whilst the rest of the blood tests were normal. Bilateral mammography and clinical exam did not yield any signs of relapse. Considering that the value of CA15-3 was over 15 times the upper limit, a total body CT scan was scheduled for April of 2019. It revealed a left retroocular relapse, bone metastases, ascites in a medium quantity and peritoneal carcinomatosis. Palliative mono-chemotherapy is initiated with Paclitaxel 175 milligrams (mg)/m2 every 3 weeks, combined with bisphosphonates: Ibandronic Acid 6 mg every 3 weeks. 

The chemotherapy was barely tolerated with several adverse reactions: grade 2 emesis on the first two cycles of chemotherapy which required antiemetic intravenous medication, febrile neutropenia after the 4th cycle which remitted after symptomatic approach with antibiotics and antipyretics. On the following cycles secondary prophylaxis with a granulocyte colony-stimulating factor was associated. During chemotherapy, the patient developed grade 3, peripheral neuropathy, and expression of the addition of chemotherapy toxicity to the underlying diabetes mellitus with unfavorable response to co-analgesics. Furthermore, at a follow up psychiatric consult, Zopiclonum 7.5 mg o.d. was added to manage the mixt insomnia. ECOG performance was elevated to 2.

After the 6th cycle of chemotherapy, patient status got worse secondary to clinical disease progression, complicated with sub-occlusive syndrome and increase in ascites quantity. The patient was transferred to the palliative care ward. Unfortunately, despite best supportive care, patient evolution was unfavorable resulting in her death in December 2019. 

## 3. Discussions

Several solid tumors and pseudo tumors can cause metastases of the eye and orbit, but breast cancer gives more frequently metastases at this level with variable incidences [4,7,8].

It is possible that the orbital metastases incidence may be higher than reported, and this underestimation could be explained by the fact of patients with orbital lesions of small dimensions are asymptomatic, or the clinical picture of ocular metastases is overshadowed by the other metastases in other organs [9]. The autopsy studies report a higher rate of ocular metastases [10]. 

In contrast to uveal metastases, which can be bilateral (up to 25%), unilateral orbital metastases are the usual presentation and the superior lateral extraconal quadrant is most frequently involved [11]. Orbital metastases are usually discovered in known breast cancer patients and they are found in the context of a multi-system end-stage disease [12]. Most reports cite that up to 25% of the total orbital metastases cases are discovered before the diagnosis of the primary tumor [7,13], as our case did.

Unlike primary orbital tumors, where the symptoms tend to onset earlier, symptoms produced by orbital metastasis include diplopia, pain, vision re-duction, proptosis, alterations of eye motility, the emergence of a tangible mass, ptosis, displacement of the globe, conjunctive chemosis, enophthalmos, papilledema, retinal folds, paresthesia, and pulsation, unlike primary orbital tumors, the symptoms tend to onset earlier. [7]. Our patient presented several of the most frequent symptoms and namely vision loss, alteration of ocular motility and proptosis in the affected eye secondary to extra-ocular muscle infiltration. 

MRI is the gold standard for evaluating orbital tumors because this ensures a better contrast of the soft tissue and does not afflict ionizing radiation. Imaging patterns such as the appearance of a mass accompanied by muscular or fatty infiltration generally suggests the presence of a mammary carcinoma metastasis, osseous affection indicates the existence of prostate cancer and prevailing muscular affliction reveals a melanoma. Mainly, orbital metastases are located exclusively in extraconal spaces (50%) and the rest are found either in intraconal spaces (30%) or in both (20%) [14]. The execution of diffusion weighted imaging can improve the differential diagnosis between an orbital process and a benign tumor (such as a hemangioma), sarcoidosis, Wegener granulomatosis and lymphoproliferative disorders. If the T2-weighted images series shows hypointense signals it may correspond to an inflammatory pseudo tumor, while hyperintense lesions may reveal a lymphoma or metastasis [14,15].

To establish a definitive diagnosis, a tissue biopsy is needed (open biopsy or fine needle aspiration). However, in a known metastatic cancer patient, a new orbital metastasis does not require a pathological confirmation [16]. The situations in which a biopsy is required are the following: patients with no known previous cancer, a solitary metastasis to the orbit with no other metastatic disease or in those cases in which decreasing tumor burden may result in a more effective chemotherapy or irradiation [17].

Breast cancer is histologically a very heterogeneous disease. ILC represents 10–15% of the total breast cancers, being, histologically, the second most frequent type of breast cancer after invasive ductal carcinoma [18]. The ILC histological subtype metastasizes in the orbitals more frequently than invasive ductal carcinoma [19]. In our patient’s case, the histopathology results of the primary breast tumor are discordant with the orbital metastasis, revealing a less common subtype of breast cancer and namely tubulo-lobular carcinoma. According to the 2012 World Health Organization (WHO) classification, tubulo-lobular carcinoma is a distinct subtype of mammary carcinoma with overlapping morphologic features of ductal and lobular invasive carcinoma. Some authors consider it a subtype of invasive ductal carcinoma, based on positivity for E-cadherin, while others claim it is a variant of invasive lobular carcinoma [20,21]. In our case, the contingent with lobular morphology and the small ducts from the breast resection specimen were negative for E-cadherin, with internal positive control on the benign glands. The presence of small ducts imposed classification as ductulo-lobular carcinoma rather than mixed, lobular and ductal carcinoma in which the ductal component had to be positive for E-cadherin. In the specimen of retroorbital metastasis excised in another hospital, there were also described small tubules/ductules and no IHC staining for E-cadherin was performed.

Considering that most metastatic breast cancers are incurable, the intent of the treatment is palliative and to improve overall survival and quality of life. The treatment of orbital metastases includes radiotherapy, hormone therapy, and systemic chemotherapy with or without target therapy. Usually, in this setting, metastasectomy is not recommended since this will not cure the disease and is associated with higher ocular morbidity [4,7,8]. In a few selected patients, as is the presented case, the excision of the tumor could establish a diagnosis [8]. External beam radiation therapy is the main treatment option for orbital and ocular adnexal metastases with an objective response rate up to 79% [22]. Despite symptom alleviation or remission [4], potential significant side effects could outweigh the benefits (cataract formation, radiation retinopathy) [8]. In the reported case, the patient developed cataract as a side effect with progressive blindness of her left eye. The gold standard treatment is still in debate. In a retrospective study by Rosset et al., it was found that 3 out of 26 patients with unilateral disease developed contralateral metastasis when only one eye was irradiated versus none of the 10 patients who received bilateral irradiation [23]. On the other hand, basing on another study by Chik et al. in which none of the 11 patients has developed contralateral metastases regardless if they received unilateral or bilateral irradiation, we consider that the only reasonable option is to treat only the affected eye and consider irradiating the contralateral eye when metastases occur [24].

Endocrine therapy should be considered the treatment of first choice for a postmenopausal metastatic breast cancer with positive estrogen receptors, regardless of the metastatic site and if a rapid tumor response is not necessary. Chemotherapy is given to patients with concomitant progressive systemic disease or if the disease has progressed on hormone therapy [25]. Although the surgical modality of primary metastatic breast cancer has a well-established role in prevention and/or treating local complication, more and more studies have shown that the regional treatment of the primary tumor, especially followed by RT, has a good impact on the prognosis with improved survival in patients with metastatic breast cancer [26,27].

The prognosis of patients with orbital metastases is poor. The median survival after diagnosis of orbital metastases from a breast cancer primary is ranging from 22 to 31 months, although survival up to 116 months has been reported [4,8]. Our patient had a progression free survival of four years with hormonotherapy. Disease recurrence was chemo refractory with taxane-based therapy. After eight only months, patient had deceased through organ insufficiency and disease progression. Overall survival of our patient was 56 months, which is longer than the median survival reported in literature. 

## 4. Conclusions

We report a case of a retroocular metastasis as the first and unique manifestation of a breast cancer and also, by our knowledge, the first case of a metastasis to the orbital region of a tubulo-lobular carcinoma. Although, orbital metastases with origins from a breast cancer, are relatively infrequent and they are associated with a reserved prognosis. Under a multidisciplinary treatment the survival of this patient has surpassed that of reported literature cases. In the case of isolated orbital metastasis, local treatment combined with systemic therapy can improve the survival rate.

Patients who present symptoms comprising of diplopia, exophthalmia, pain, ptosis, or clinically detected breast tumor should be investigated for orbital metastasis even in the absence of history of cancer. Being responsible for the majority of ocular/orbital metastasis, breast cancer must be taken into consideration as the most likely cause. Clinical and paraclinic investigations of the breast may identify the primary neoplasia.

## Data Availability

The data presented in this study are available on request from the corresponding author. The data are not publicly available in order to limit the amount of publicly-available patient personal information, as classified by the European Union General Data Protection Regulation.
